# Prospective planning comparison of magnetic resonance-guided vs. internal target volume-based stereotactic body radiotherapy of hepatic metastases – Which patients do really benefit from an MR-linac?

**DOI:** 10.1016/j.ctro.2025.100941

**Published:** 2025-02-28

**Authors:** Philipp Hoegen-Saßmannshausen, C. Katharina Renkamp, Hoi Hin Lau, David Neugebauer, Nina Niebuhr, Carolin Buchele, Fabian Schlüter, Elisabetta Sandrini, Line Hoeltgen, Fabian Weykamp, Sebastian Regnery, Jakob Liermann, Eva Meixner, Kevin Zhang, Oliver Sedlaczek, Heinz-Peter Schlemmer, Laila König, Jürgen Debus, Sebastian Klüter, Juliane Hörner-Rieber

**Affiliations:** a Department of Radiation Oncology, Heidelberg University Hospital, Heidelberg, Germany; b Heidelberg Institute of Radiation Oncology (HIRO), Heidelberg, Germany; c National Center for Tumor Diseases (NCT), Heidelberg, Germany; d Clinical Cooperation Unit Radiation Oncology, German Cancer Research Center (DKFZ), Heidelberg, Germany; e Department of Radiation Oncology, RKH Klinikum Ludwigsburg, Ludwigsburg, Germany; f Division of Radiology, German Cancer Research Center (DKFZ), Heidelberg, Germany; g Department of Radiation Oncology, Heidelberg Ion Beam Therapy Center (HIT), Heidelberg University Hospital, Heidelberg, Germany; h German Cancer Consortium (DKTK), Partner Site Heidelberg, Heidelberg, Germany; i Department of Radiation Oncology, Düsseldorf University Hospital, Düsseldorf, Germany

**Keywords:** SABR, SMART, Online adaptive radiotherapy, Oligometastasis, Oligoprogression, Liver metastases

## Abstract

**Purpose/objective:**

To compare online MR-guided SBRT (MRgRT) of liver metastases with state-of-the-art ITV-based SBRT (ITV-SBRT) and assess which patients benefit most from MRgRT.

**Materials and methods:**

In a prospective randomized trial (MAESTRO study, NCT05027711), patients were randomized to either gated and online adaptive MRgRT or ITV-SBRT if a biologically effective dose (BED_10_) of 100 Gy was feasible with ITV-SBRT. Otherwise, patients were treated with MRgRT. In this subgroup analysis of 20 patients, a dosimetric comparison of MRgRT and ITV-SBRT plans was performed. Tumor control and normal tissue complication probabilities were calculated.

**Results:**

In 40 % of all patients, MRgRT enabled SBRT with less fractions and/or higher prescription BED_10_. Almost all target volume metrics were improved with MRgRT. MRgRT PTV D95% was significantly higher in the overall cohort (91.0 ± 22.9 Gy vs. 79.5 ± 27.2 Gy, p = 0.001), in uncritical (111.3 ± 6.2 Gy vs. 104.7 ± 4.1 Gy, p = 0.022) and in critical cases with limited healthy liver volume or nearby gastrointestinal organs at risk (74.1 ± 16.9 Gy vs. 58.5 ± 18.5 Gy, p = 0.041). Target volume V100% was also superior with MRgRT. Calculated 2-year tumor control probability was significantly superior with MRgRT overall (73.0 ± 6.2 % vs. 69.7 ± 7.9 %, p = 0.002), in uncritical cases (78.3 ± 1.4 % vs. 76.8 ± 1.0 %, p = 0.022) and in critical cases (68.5 ± 4.8 % vs. 63.8 ± 5.8 %, p = 0.041), without elevated normal tissue complication probability.

**Conclusion:**

Dosimetrically, gated MRgRT was beneficial for virtually all the hepatic metastases analyzed in this study. Patients with metastases located critically near gastrointestinal OAR or with limited healthy liver volume should be allocated to centers providing MRgRT.

## Highlights

• This is the first comparison of gated MR-guided and state-of-the-art ITV-based SBRT.
• In 8 of 20 patients, MRgRT enabled less fractions and/or higher prescription BED_10_.
• PTV D95% and V100% were significantly higher with MRgRT.
• Tumor control probability was significantly superior with MRgRT.

## 1 Introduction

Recently, several studies have established the use of stereotactic body radiotherapy (SBRT) in patients with oligometastases [1], [2], [3] and oligoprogression [4]. SBRT enables effective treatment for both liver metastases and hepatocellular carcinoma (HCC) [5], [6]. A widely used technique is SBRT based on an internal target volume (ITV) to compensate for tumor motion [7]. Inherently, breathing motion and ITV overlap with nearby radiosensitive organs at risk (OAR) frequently cause constraint violations and necessitate reduction of the prescribed target volume doses.

Further, conventional non-adaptive SBRT is limited by poor soft tissue contrast in CBCT and the impossibility to consider inter-fractional variations [8].

Technically, the introduction of MR-linacs has enabled online-adaptive MR-guided radiotherapy (MRgRT). With superior soft tissue contrast, online plan adaptation, live imaging during dose delivery and small planning target volume (PTV) margins enabled by gating, this technique has demonstrated its ability to mitigate interfractional anatomical changes [9], [10], [11].

Even in developed countries, advanced techniques like particle therapy, robotic radiotherapy, or MRgRT may be limited. Dedicated techniques are more expensive than standard linear accelerators. In online-adaptive radiotherapy, physicians and physicists must be on-site throughout the adaptation process [12]. Additional costs are often not covered by public health insurance funds so far, e.g. in Germany. Given the limited financial resources in many public health systems, public institutions and health funds increasingly demand randomized trials demonstrating the benefit of new techniques [13].

Currently, it remains unclear which patients benefit most from MRgRT and should be allocated to dedicated centers. To the best of our knowledge, no prospective studies have compared hepatic ITV-based SBRT (ITV-SBRT) and MRgRT. Available planning comparisons between MRgRT and other techniques have important limitations: either they did not use the actual treatment planning software and beam models of available MR-linacs [14], [15], or margins between compared techniques deviated largely [16].

Thus, a comparison of real-world, clinically deliverable plans for hepatic MRgRT with a widely available technique such as state-of-the-art ITV-SBRT is still pending. The prospective, randomized MAESTRO study (NCT05027711) seeks to fill this void [17].

## 2 Materials and Methods

### 2.1 Patients

The first 20 patients within the MAESTRO trial treated with MRgRT were included in the current analysis. Details of the trial have been published before [17]. In brief, patients with up to three active liver metastases (maximum single GTV diameter 5 cm, cumulative up to 12 cm for three metastases) were enrolled. Initially, all patients received a treatment simulation CT for ITV-SBRT. If a biologically effective dose for α/β = 10 Gy (BED_10_) of 100 Gy (with a PTV coverage of 95 % to the surrounding isodose) was feasible, patients were randomized between ITV-SBRT and MRgRT. Otherwise, a clear benefit of MRgRT was assumed and patients were treated accordingly without randomization. The present analysis comprises the first 20 patients with treatment plans for both ITV-SBRT and MRgRT, i.e. either randomized directly to MRgRT (study arm A, n = 10) or treated with MRgRT due to a BED_10_ < 100 Gy (study arm C, n = 10). Patients randomized to study arm B (ITV-SBRT) did not receive MRgRT simulation and were excluded from the present analysis. The study was designed according to the declaration of Helsinki. Patients were prospectively enrolled after written informed consent (institutional ethics board approval S-002/2021).

### 2.2 Treatment simulation, planning and prescription

All patients received a diagnostic 1.5 T MRI on a single scanner (1.5 T, Siemens Magnetom Aera, Siemens Healthineers, Erlangen, Germany) using standard multichannel body coils and integrated spine phased-array coils. MRI protocol included the following diagnostic sequences: (a) coronal T2-weighted, single-shot fast-spin echo sequence (HASTE; repetition time (TR)/echo time (TE) 1000 ms/67 ms, voxel size (VS) 0.6 × 0.6 × 5 mm); (b) T2-weighted, fat-saturated single-shot fast-spin echo sequence (HASTE, TR/TE 1200 ms/91 ms, VS 1.2 × 1.2 × 5 mm); (c) axial T1-weighted, 3D gradient-echo dixon sequence (VIBE; TR/TE 6.69 ms/2.39 ms − 4.77 ms, VS 0.6 × 0.6 × 3 mm); (d) single-shot echo planar diffusion weighted imaging (b-values: 50, 800–900; TR/TE 3900–8400 ms/58–59 ms; VS 2.0–2.3 × 2.0–2.3 × 5.0–6.0 mm); (e) T1-weighted native and dynamic contrast-enhanced 3D gradient-echo dixon sequence (VIBE, TR/TE 6.7 ms/2.39 ms-4.77 ms, VS 1.2 × 1.2 × 3.0 mm) or free-breathing, fat-saturated GRASP sequence (GRASP VIBE, TR/TE 4.2 ms/2.35 ms, VS 1.5 × 1.5 × 3.0 mm); (f) coronal T1-weighted, fat-saturated 3D gradient-echo sequence (VIBE; TR/TE 5.1 ms/1.87 ms, VS 0.6 × 0.6 × 3 mm).

For ITV-SBRT, patients were immobilized with vacuum cushions and abdominal compression, if tolerated. Simulation CT included contrast-enhanced scans and a 4D-CT with eight phases to assess tumor and OAR motion.

For MRgRT, a 3D TrueFISP sequence was acquired in inspiration breath-hold without abdominal compression (axial resolution 1.5 × 1.5 mm^2^ and slice thickness 3 mm). One to three 2D CINE sequences (2–8 frames per second) were used for gating. For dose calculation, a non-contrast-enhanced CT was acquired in treatment position unless the initial simulation CT could be used. In any case, dose calculation was based on the acquisition of a real CT and not on the creation of a synthetic CT.

Gross tumor volumes (GTV) were delineated using diagnostic MRI and simulation CT for ITV-based SBRT and, additionally, 3D TrueFISP MRI for MRgRT. For ITV-SBRT, an ITV was generated by contouring the GTV on all 4D images. The clinical target volume (CTV) was created by adding a margin of 5 mm to the GTV or ITV, respectively, limited to the liver. Finally, a PTV margin of 5 mm (ITV-SBRT) or 3 mm (MRgRT) was added. For gastrointestinal OAR close to the PTV, a planning organ at risk volume (PRV) was created, comprising the respective OAR on all 4d-CT images [18].

Dose was prescribed to the surrounding 65–80 % isodose, with an aspired PTV coverage of 95 % (V100% ≥95 %). Violation of OAR constraints (shown in Table 1) was not permitted. If adequate PTV coverage at the prescribed dose was not possible, prescription dose had to be lowered up to 10 fractions of 5.0 Gy (BED_10_ = 75.0 Gy). If a PTV D95% of 100 % was still not possible, lower PTV coverage was accepted.

**Table 1:** Organ at risk (OAR) constraints for prescription regimen used in the study.

		Dose constraint [Gy]			
OAR	Metric	3 fractions	5 fractions	8 fractions	10 fractions
Liver- CTV	D(volume-700 cc)	< 19.2	< 24.0	< 29.0	< 32.0
Esophagus	D0.5 cc	<25.2	<34.0	<40.0	< 43.5
Stomach	D0.5 cc	<22.2	<35.0	<40.0	< 43.5
Duodenum	D0.5 cc	<22.2	<35.0	<40.0	< 43.5
Bowel	D0.5 cc	<25.2	<35.0	<40.0	< 43.5
Kidneys (individual)	D_mean_	<8.5	<10.0	<11.5	<12.0
Heart	D0.5 cc	<25.0	<29.0	<60.0	<66.0
Spinal cord	D0.1 cc	<21.6	<27.0	<32.0	<35.0

For ITV-SBRT, contouring and planning was performed in RayStation (Version 11B, RaySearch Laboratories AB, Stockholm, Sweden). Plans were generated as volumetric arc therapies (VMAT) for Elekta VersaHD and Synergy linacs with matched beams (Elekta AB, Stockholm, Sweden). MRgRT contouring was performed in RayStation and ViewRay treatment planning systems (TPS) for the MRIdian Linac® (ViewRay Inc., Denver, USA). MRgRT plans were gated step-and-shoot IMRT plans with variable amounts of beams and segments.

### 2.3 Plan evaluation

GTV, CTV and PTV were compared with regard to mean doses, doses at X% volume (DX%) and volumes at Y% dose (VY%). All GTV, CTV and PTV doses were transformed to BED_10_:

$BED10=n*d\;(1+\frac{d}{10})$

with n fractions and fraction dose d. Tumor control probability (TCP) was calculated as

$$TCP\left(d\right)=\frac{100\%}{1+exp(\frac{TCD50-d}{k})}$$

with dose d (as BED_10_), TCD_50_ as the dose achieving a tumor control of 50 %, and a fitting constant k [19]. TCD_50_ = 16 Gy and k = 74 Gy were chosen according to Ohri et al. for 2-year TCP [20].

PTV conformity indices (CI) were computed as [21]

$$\mathrm{CI}=\frac{{\mathrm{PTV}(PIV)}^{2}}{\mathrm{PTV}*PIV}$$

with PIV as the total volume covered by the prescription isodose and PTV(PIV) as the PTV covered by the prescription isodose.

Normal tissue complication probabilities (NTCP) for liver and gastrointestinal (GI) tract were calculated with the Lyman-Kutcher-Burman model [22], [23], [24] similarly to a previous study [15], with the use of various established models:

1. Liver: radiation-induced-liver disease (RILD) >= grade 3: TD_50_ = 43.3, n = 1.1, m = 0.18, D_ref_ = 1.5 Gy, α/β = 2.0 Gy [25]
2. Liver: increase of albumin-bilirubin (ALBI) grade [26] >= 1: TD_50_ = 32, n = 2, m = 1.5, D_ref_ = 2.0 Gy, α/β = 2.5 Gy [27]
3. Liver: increase of Child-Pugh (CP) score >= 2: TD_50_ = 19, n = 16.67, m = 0.8, D_ref_ = 2.0 Gy, α/β = 2.5 Gy [27]
4. GI tract: gastric bleeding: TD_50_ = 180, n = 0.12, m = 0.49, D_ref_ = 2.0 Gy, α/β = 2.5 Gy [28]
5. GI tract: duodenal toxicity >= grade 3: TD_50_ = 299.1, n = 0.193, m = 0.51, D_ref_ = 2.0 Gy, α/β = 4.0 Gy [29]
6. GI tract: duodenal toxicity grades 2–4: TD_50_ = 24.6, n = 0.12, m = 0.23, D_ref_ = 25.0 Gy, α/β = 4.0 Gy [30].

First, OAR doses were converted to equivalent doses for the reference dose of the respective model [15]:

$$EQD\left(Dref\right)=D\left(\frac{d+\alpha /\beta }{D\;ref+\alpha /\beta }\right)$$

With this EQD_D ref_ as dose D, the equivalent uniform dose EUD was calculated for all partial volumes V_i_ with dose D_i_ [31]:

$$\mathrm{EUD}={\left(\sum_{i}D_{i}^{\frac{1}{n}}\frac{V_{i}}{V_{\mathrm{tot}}}\right)}^{n}$$

NTCP [32] was calculated as:

$$\mathrm{NTCP}=\frac{1}{2\pi }\int_{-\infty }^{t}e^{\frac{{-t}^{2}}{2}}dt$$

with $t=\frac{\left(EUD-{\mathrm{TD}}_{50}\right)}{m*{\mathrm{TD}}_{50}}$

Simulation imaging for ITV-SBRT and MRgRT was performed at different dates. Variable filling of hollow organs and the use of abdominal compression for ITV-SBRT could lead to different anatomy, position and PTV proximity of OAR. As we sought to focus on the dosimetric differences attributable to SBRT technique, the potentially dose-limiting OAR stomach, duodenum, and bowel were summarized as “GI tract”.

### 2.4 Statistical analysis

Statistical analysis was performed using SPSS version 29 (IBM Corporation, Armonk, NY, USA). Wilcoxon Rank test was used for comparison of dependent, continuous, not normally distributed data. P-values < 0.05 were considered statistically significant. In box plots, dots represent outliers and stars represent extreme values.

## 3 Results

### 3.1 Patient and treatment characteristics

Of the ten patients with an ITV-SBRT BED_10_ < 100 Gy, the dose-limiting OAR were stomach/duodenum/bowel in eight cases and healthy liver in two cases (in one patient, both GI tract and liver were dose-limiting). In one patient, tumorous infiltration of the right kidney was dose-limiting. Of the eight patients with GI tract as dose-limiting OAR, seven had subcapsular liver metastases. In the eight one, distance between GTV and liver capsule was about 1 cm. As GI tract and healthy liver were the most common dose-limiting OAR, these were investigated further with NTCP modelling.

Patient characteristics are shown in Table 2. GTV, CTV and PTV volumes were 18.6 ± 38.7 cc, 49.9 ± 57.2 cc and 97.8 ± 80.7 cc for ITV-SBRT and 23.8 ± 44.0 cc, 46.6 ± 60.6 cc and 67.5 ± 73.4 cc for MRgRT. Respective p-values were p < 0.001, p = 0.171 and p < 0.001.

**Table 2:** Patient characteristics.

	Whole cohort n = 20	Study arm A BED_10_ >= 100 Gy with ITV-SBRT n = 10	Study arm C BED_10_ < 100 Gy with ITV-SBRT n = 10
Amount of liver metastases			
1	16	8	8
2	1	1	0
3	3	1	2
Affected liver segments			
II	4	0	4
II/III	3	3	0
III	1	0	1
IV	1	0	1
IVa	2	1	1
IVb	4	0	4
VI	3	3	0
VII/VIII	2	1	1
VII	3	2	1
VIII	4	3	1
Abdominal compression			
Yes	15 (75 %)	7 (70 %)	8 (80 %)
No	5 (25 %)	3 (30 %)	2 (20 %)
Planning organ at risk volume (PRV)			
Yes	12 (60 %)	4 (40 %)	8 (80 %)
No	8 (40 %)	6 (60 %)	2 (20 %)

**Fig. 1:**
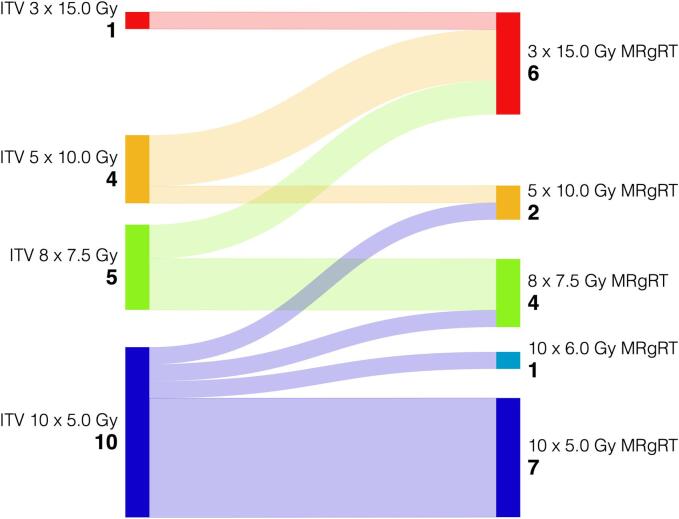
Prescription doses (to the surrounding isodose) and respective number of patients with ITV-SBRT (left) and MRgRT (right). Figure created with SankeyMATIC.com.

In eight patients, MRgRT enabled a regimen with fewer fractions and/or higher BED_10_, displayed in Fig. 1. This included five patients of Arm A treated with fewer fractions, two patients of Arm C treated with fewer fractions and a BED_10_ of >= 100 Gy and one patient of Arm C treated with a higher BED_10_ in ten fractions.

### 3.2 Target volume dosimetry

**Fig. 2:**
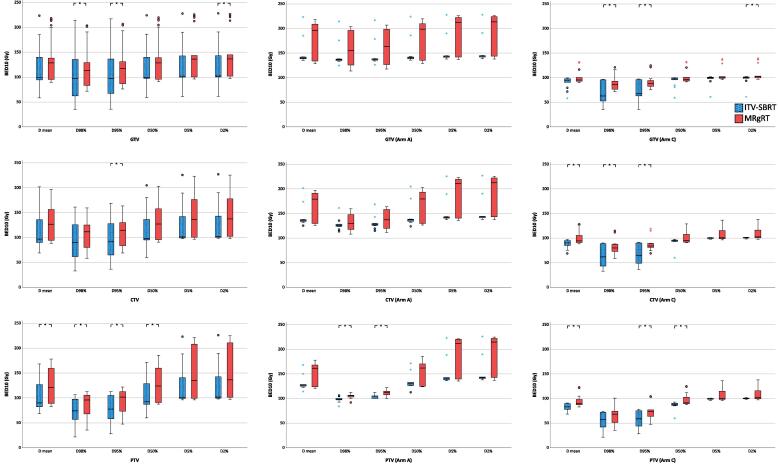
Dose metrics with ITV-SBRT (blue) and MRgRT (orange) as BED_10_ for GTV (upper line), CTV (middle line) and PTV (lower line) in the whole cohort (left column), in study arms A (middle column) and C (right column). * represent p-values < 0.05. (For interpretation of the references to colour in this figure legend, the reader is referred to the web version of this article.)

For most GTV, CTV and PTV metrics evaluated, the median BED_10_ was higher with MRgRT (Fig. 2). In particular, PTV D95% was significantly superior with MRgRT in the overall cohort and both study arms, with 79.5 ± 27.2 Gy (ITV-SBRT) vs. 91.0 ± 22.9 Gy (MRgRT) overall (p = 0.001), 104.7 ± 4.1 Gy vs. 111.3 ± 6.2 Gy in arm A (p = 0.022) and 58.5 ± 18.5 Gy vs. 74.1 ± 16.9 Gy in arm C (p = 0.041).

**Fig. 3:**
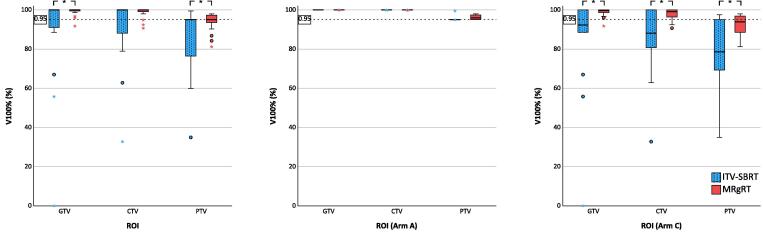
Target volume V100% with ITV-SBRT (blue) and MRgRT (orange) in the whole cohort (left), in study arms A (middle) and C (right). * represent p-values < 0.05. (For interpretation of the references to colour in this figure legend, the reader is referred to the web version of this article.)

Target volume V100% was superior with MRgRT (significant for GTV and PTV). In study arm A, V100% did not differ significantly between ITV-SBRT and MRgRT, while difference was significant for GTV, CTV and PTV in study arm C (Fig. 3). Here, the use of MRgRT instead of ITV-SBRT improved GTV V100% from 84.0 ± 27.6 % to 98.6 ± 2.4 % (p = 0.011), CTV V100% from 84.9 ± 2.0 % to 97.7 ± 3.2 % (p = 0.021) and PTV V100% from 78.7 ± 19.0 % to 92.3 ± 5.5 % (p = 0.012).

**Fig. 4:**
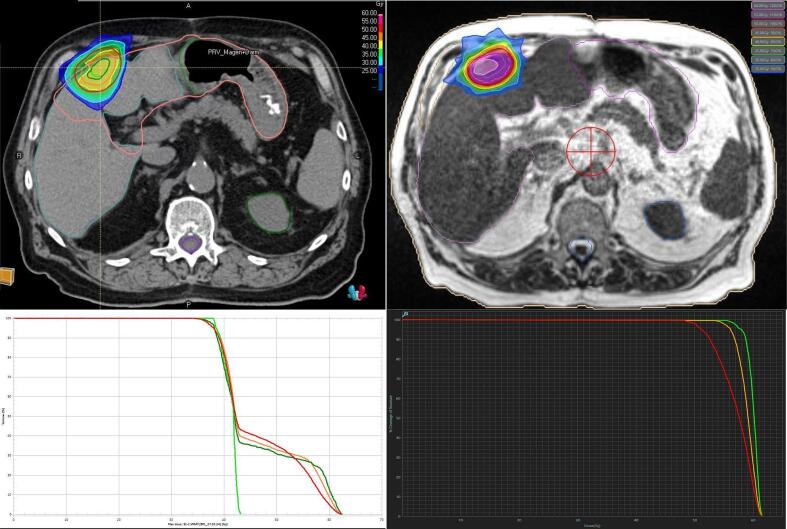
Dose distribution and dose volume histograms (DVH) with ITV-SBRT (left) and MRgRT (right) in a patient with overlap of GTV and gastrointestinal PRV in ITV-based planning. DVH lines: light green = GTV, dark green = ITV, orange = CTV, red = PTV. (For interpretation of the references to colour in this figure legend, the reader is referred to the web version of this article.)

Detailed p-vales for all comparisons are shown in Supplement 1. Conformity indices are displayed in Table 3. Fig. 4 illustrates dose distributions in a patient with GTV-PRV overlap in ITV-SBRT*.*

**Table 3:** Conformity indices with ITV-SBRT and MRgRT in the overall cohort and in study arms A and C.

	ITV-SBRT	MRgRT	p-value
Whole cohort	0.80 ± 0.18	0.85 ± 0.06	0.548
Study arm A	0.92 ± 0.03	0.87 ± 0.04	0.007*
Study arm C	0.71 ± 0.20	0.83 ± 0.07	0.019*

### 3.3 Normal tissue complication and tumor control probabilities

NTCP for healthy liver and GI tract are shown in Table 4. TCP based on the PTV D95% is displayed in Table 5. MRgRT led to a mean TCP increase of 3.3 % in the overall cohort, 1.5 % in arm A and 4.7 % in arm C.

**Table 4:** Normal tissue complication probability (NTCP) for healthy liver (liver-CTV) and gastrointestinal tract (GI tract, = stomach/duodenum/bowel) calculated with various models for ITV-SBRT and MRgRT.

OAR	Model Author	Endpoint	ITV-SBRT		MRgRT		p-value
			Mean ± StDev	Max	Mean ± StDev	Max	
Liver	Dawson [25]	RILD >= grade 3	2.2 ± 4.2 %	15.0 %	1.4 ± 3.5 %	13.3 %	0.057
	Pursley [27]	Albumin-bilirubin increase grade 1 or 2	14.3 ± 0.9 %	15.8 %	14.0 ± 0.8 %	15.6 %	0.004*
	Pursley [27]	Child-Pugh score increase >= 2	11.3 ± 3.0 %	15.9 %	10.3 ± 2.4 %	15.6 %	0.030*
GI tract	Pan [28]	Gastric bleeding	3.1 ± 0.8 %	5.0 %	3.0 ± 0.7 %	4.6 %	0.263
	Holyoake [29]	Duodenal toxicity >= grade 3	2.7 ± 0.3 %	3.4 %	2.7 ± 0.2 %	3.1 %	0.167
	Murphy [30]	Duodenal toxicity grade 2–4	0.0 ± 0.1 %	0.3 %	0.0 ± 0.0 %	0.2 %	0.191

**Table 5:** Estimated tumor control probability at two years with ITV-SBRT and MRgRT in the overall cohort and in study arms A and C.

	ITV-SBRT	MRgRT	p-value
Whole cohort	69.7 ± 7.9 %	73.0 ± 6.2 %	0.002*
Study arm A	76.8 ± 1.0 %	78.3 ± 1.4 %	0.022*
Study arm C	63.8 ± 5.8 %	68.5 ± 4.8 %	0.041*

## 4 Discussion

To the best of our knowledge, only one previous study has compared plans of ITV-based photon SBRT and MRgRT, albeit in a very small cohort. This study used a PTV margin difference of 5 mm between the two techniques [16], which is very likely to overlay all other aspects related to SBRT technique. Thus, the present study is the first one comparing deliverable plans of state-of-the-art ITV-SBRT with small margins and MRgRT in a prospective setting and larger cohort.

Hepatic metastases should be treated with a BED_10_ >= 100 Gy for satisfactory local control [20], [33], [34]. Thus, a PTV BED_10_ of 100 Gy has been chosen as threshold for randomization in this prospective study. In comparison to ITV-SBRT, MRgRT enabled more aggressive dose prescription and fractionation. Half of all patients in arm A could be treated with fewer fractions. In 30 % of patients that could only be treated with a prescribed BED_10_ of 75 Gy with ITV-SBRT, MRgRT allowed for a prescription BED_10_ of 96 Gy or higher. The present analysis covered all relevant target volume metrics for SBRT, including mean dose, D98%, D95%, D50%, D5% and D2% for GTV, CTV and PTV [35]. Most of these metrics could be improved by employing MRgRT. In particular, GTV, CTV and PTV D95% were significantly higher with MRgRT. PTV D95% was significantly higher with MRgRT in both subgroups, while GTV and CTV D95% were only significantly higher in critical cases (study arm C). Yoda et al. reported PTV D95% BED_10_ improvements of 11.0 – 45.7 Gy in five patients, albeit in comparison to unjustifiably large ITV-PTV margins [16]. In the current setting with more reasonable margins, mean PTV D95% BED_10_ increase was 10.5 Gy overall, 6.6 Gy in uncritical cases and 15.6 Gy in critical cases. GTV and PTV V100% were also significantly higher with MRgRT in the whole cohort, but subgroup analysis showed that this was mainly driven by the large improvements in study arm C.

Resulting TCP was significantly superior with MRgRT in the whole cohort and both subgroups: even for metastases that could have been treated with a potentially sufficiently high dose using ITV-SBRT, MRgRT led to a significant improvement in PTV D98% and PTV D95%. For metastases in critical proximity to gastrointestinal OAR or in cases with a limited healthy liver volume, the effect is highly significant and clinically relevant, as mean TCP could be improved by 4.7 %.

Large multi-center studies reported two-year local control rates of 63.8 % [34] and 75 % [36]. If stratified for BED_10_, two-year local control was 59.6–70 % (BED_10_ < 100 Gy) or 77.2–93 % (BED_10_ >= 100 Gy) [20], [37]. All in all, the two-year TCP calculated here and the difference between the study arms are in accordance with published data.

Gastrointestinal NTCP was low and did not differ significantly between ITV-SBRT and MRgRT. As per-protocol OAR constraints had to be respected and PRVs accounted for OAR motion in ITV-SBRT, comparably low NTCP were achieved with both techniques. Healthy liver complication probabilities of elevated ALBI and Child-Pugh scores were significantly lower with MRgRT, but with about 1 % not to a degree we would classify as clinically relevant. NTCP published in other works show large ranges and patient-specific differences [14], [15], [38]. In accordance with these studies, the risk for less critical liver enzyme elevation is higher than the more severe risk of RILD.

Conformity indices did not differ significantly in the overall cohort. However, the two study arms showed significant differences in opposite directions: in arm A, ITV-SBRT plans were more conformal, while MRgRT plans were more conformal in arm C. In uncritical cases, the superior VMAT technique applied with ITV-SBRT compared to only step-and-shoot IMRT with MRgRT comes into play. In critical cases with radiosensitive gastrointestinal OAR nearby, however, higher dose conformity to the target volume can be achieved with gated MRgRT.

Comparison of contours in the two different TPS revealed that GTV volumes were larger in MRgRT plans. As a first explanation, even 0.35 T TRUFI images add additional MRI-specific imaging information with superior soft tissue contrast compared to CT-based planning, without the limitations of prior image registration inherent to secondary diagnostic MR images. Second, median time interval between ITV-SBRT simulation and MRgRT simulation was 11 days (maximum 21 days). In patients with fast tumor growth, this might explain a GTV volume difference. Finally, volume definition may also vary between different TPS. CTV and PTV volumes were larger with ITV-SBRT, showing the benefit of gating and online image guidance instead of an ITV.

The two commercially available MR-linac systems have been compared in detail in various articles [8], [39]. The specific difference probably most relevant with regard to the present study is that, until recently, automated gating was not available at the 1.5 T MR-linac, but had to be performed visually and manually [8], [40]. Recently, this limitation could be overcome [8], [39], [41]. On the other hand, the 1.5 T MR-linac offers a broader variety of MR sequences and superior MR image quality due to the higher field strength [8].

Compared to photon MR-linacs, proton and carbon ion therapy, potentially MR-guided, could further reduce OAR doses, especially to the healthy liver [14], [15], [42]. Drawbacks of proton and carbon ion therapy include the limited availability, even in comparison to MR-linacs, and susceptibility to motion referred to as “interplay effect” [43], [44].

Two limitations related to the study design should be noted. First, a clear benefit for MRgRT was assumed a priori in the study arm C. However, as BED_10_ did not reach the levels required for optimal local control, we considered it unethical to treat these patients with ITV-SBRT, given that MRgRT was available on-site. Second, no MRgRT simulation images were acquired in study arm B, thus prohibiting inclusion of this study arm in the present analysis. Furthermore, simulation imaging for ITV-SBRT and MRgRT at different dates may also have caused minor anatomic deviations, potentially influencing the resulting plans.

The demonstrated superiority of MRgRT is probably partially attributable to the use of gating and the omission of an ITV. The beneficial effect of daily adaptation has been demonstrated before [9], [10], [11] and was not part of the present analysis. Dosimetric benefits in comparison to ITV-SBRT might also be achieved with robotic or surface-guided SBRT. However, fiducials needed for robotic SBRT are implanted invasively, may dislocate, and remain an indirect surrogate of the actual GTV [45]. Surface-guidance and inspiration breath-hold reduce target volumes compared to ITV-SBRT [46]. However, this technique may not be suitable for all liver lesions and should be handled with care, as internal deviations in the range of 1 cm can occur while the body surface is matched and as a linear correlation between body surface and liver tumor motion have been present in only half of all patients [47], [48]. In surface-guided deep inspiration breath-hold SBRT, PTV margins of 5–10 mm are common [47]. For robotic liver SBRT, proposed PTV margins range from 3-6 mm [45], [49], [50]. The 3 mm PTV margin for MRgRT commonly applied in our institution was established based on in-house research on uncertainties, especially with regard to geometric distortions in MRI, isocenter differences between MRI and linac, multi-leaf collimator positioning errors, volume effects due to voxel size and tracking/gating uncertainties [51], [52]. A 3 mm margin for gated MRgRT is in line with published data from other institutions [53], [54].

Generally, margin concepts vary between institutions. Some do not use a CTV in hepatic MRgRT, but apply PTV margins between 2 and 5 mm [16], [54]. Others use a CTV, be it in MRgRT [11] or other highly conformal techniques such as robotic SBRT [50]. Application of a different margin concept might possibly have altered the results.

Dose-response of liver metastases varies for different primaries [34], [36], [37], [55]. Higher α/β values of 16–28 Gy have been suggested [56]. However, most institutions still employ an α/β of 10 Gy [20], [36], [37].

To the best of our knowledge, no DVH-based TCP model has been established for hepatic metastases. We chose the prescription dose-based model proposed by Ohri et al. [20] because of its larger data pool compared to other models [33]. PTV prescription may vary in different publications – some authors used surrounding isodose lines, while others used isocenter dose. However, according to Ohri et al., adjusting for different prescription styles did not significantly change the modelling outcomes. As PTV D95% was the primary prescription in the present study and thus a primary goal for plan optimization, it was used for TCP calculation.

For DVH-based NTCP calculation, cropping of maximum doses to near-maximum doses has been proposed to account for the single voxel sensitivity of EUD calculations [57]. However, contouring was based on different primary images. This will usually result in different volumes of a given OAR in the two plans. Percental cropping of different structure volumes in ITV-SBRT and MRgRT would have led to different absolute partial volumes neglected, possibly falsifying analysis results. Thus, absolute maximum doses were included in the analysis. Therefore, the NTCP values reported are higher (potentially overestimating NTCP risk) compared to analyses based on near-maximum doses. Nevertheless, absolute NTCP risks reported were generally low.

Finally, this work is a dosimetric, in silico planning comparison, with all inherent limitations. Patient-specific characteristics and treatment-related events may lead to different in vivo outcomes for both TCP and NTCP. The results of the present analysis remain to be confirmed by the final clinical outcomes of this prospective study.

## 5 Conclusion

In terms of dosimetry, gated MRgRT was beneficial for virtually all the hepatic metastases analyzed in this study. In half of the uncritically located cases, MRgRT enabled SBRT in fewer fractions and/or higher BED10. For critically located metastases near gastrointestinal OAR and for patients with limited healthy liver volume, there was a significant and potentially clinically relevant benefit from MRgRT. Patients should be allocated to centers providing MRgRT with regard to these two factors. These dosimetric findings will have to be validated by prospective outcome and toxicity data.

## CRediT authorship contribution statement

**Philipp Hoegen-Saßmannshausen:** Conceptualization, Data curation, Formal analysis, Funding acquisition, Investigation, Methodology, Validation, Visualization, Writing – original draft. **C. Katharina Renkamp:** Investigation, Methodology, Software, Writing – review & editing. **Hoi Hin Lau:** Investigation, Software, Writing – review & editing. **David Neugebauer:** Investigation, Software, Writing – review & editing. **Nina Niebuhr:** Investigation, Methodology, Software, Writing – review & editing. **Carolin Buchele:** Conceptualization, Investigation, Methodology, Software, Writing – review & editing. **Fabian Schlüter:** Investigation, Writing – review & editing. **Elisabetta Sandrini:** Investigation, Writing – review & editing. **Line Hoeltgen:** Investigation, Writing – review & editing. **Fabian Weykamp:** Investigation, Writing – review & editing. **Sebastian Regnery:** Investigation, Writing – review & editing. **Jakob Liermann:** Investigation, Writing – review & editing. **Eva Meixner:** Investigation, Writing – review & editing. **Kevin Zhang:** Conceptualization, Investigation, Writing – review & editing. **Oliver Sedlaczek:** Conceptualization, Investigation, Writing – review & editing. **Heinz-Peter Schlemmer:** Conceptualization, Resources, Supervision, Writing – review & editing. **Laila König:** Investigation, Writing – review & editing. **Jürgen Debus:** Conceptualization, Funding acquisition, Resources, Supervision, Writing – review & editing. **Sebastian Klüter:** Conceptualization, Funding acquisition, Methodology, Supervision, Writing – review & editing. **Juliane Hörner-Rieber:** Conceptualization, Funding acquisition, Investigation, Methodology, Supervision, Validation, Writing – review & editing.

## Declaration of competing interest

The authors declare the following financial interests/personal relationships which may be considered as potential competing interests: PHS received compensation for advisory boards from Novocure GmbH. FW received speaker fees from AstraZeneca, Varian Medical Systems and Merck Sharp & Dohme and travel support for attending meetings from AstraZeneca, Varian Medical Systems, Novocure GmbH, Fraunhofer MEVIS and Micropos Medical as well as compensation for advisory boards from Novocure GmbH and Merck Sharp & Dohme. JL received speaker fees from Accuray Incorporated and travel fees from Micropos Medical and RaySearch Laboratories outside the submitted work. JD received grants from RaySearch Laboratories AB, Vision RT Limited, Merck Serono GmbH, Siemens Healthcare GmbH, PTW-Freiburg Dr. Pychlau GmbH and Accuray Incorporated outside the submitted work. JHR and SK received speaker fees from ViewRay Inc. JHR received speaker fees from Pfizer Inc., Sanofi, AstraZeneca and Accuracy International Sàrl as well as grants from IntraOP Medical and Varian Medical Systems outside the submitted work.
