# Supplementary material for: Prospective planning comparison of magnetic resonance-guided vs. internal target volume-based stereotactic body radiotherapy of hepatic metastases – Which patients do really benefit from an MR-linac?
Source: Clin Transl Radiat Oncol. 2025 Feb 28;52:100941. doi: 10.1016/j.ctro.2025.100941 (PMC11926716; doi:10.1016/j.ctro.2025.100941)
Supplement: Supplementary Data 1 [file mmc1.docx]

Supplement 1: p-values for all evaluated GTV, CTV and PTV metrics

| ROI | Parameter | Whole cohort | Arm A | Arm C |
| --- | --- | --- | --- | --- |
| GTV | Mean | Ns | Ns | Ns |
|  | D98% | 0.045 | Ns | 0.016 |
|  | D95% | 0.030 | Ns | 0.016 |
|  | D50% | Ns | Ns | Ns |
|  | D5% | Ns | Ns | Ns |
|  | D2% | 0.030 | NS | 0.022 |
|  | V100% | 0.016 | NS | 0.011 |
| CTV | Mean | NS | NS | 0.034 |
|  | D98% | NS | NS | 0.028 |
|  | D95% | 0.024 | NS | 0.023 |
|  | D50% | NS | NS | NS |
|  | D5% | NS | NS | NS |
|  | D2% | NS | NS | NS |
|  | V100% | NS | NS | 0.021 |
| PTV | Mean | 0.006 | NS | 0.003 |
|  | D98% | 0.004 | 0.005 | NS |
|  | D95% | 0.001 | 0.022 | 0.041 |
|  | D50% | 0.016 | NS | 0.003 |
|  | D5% | NS | NS | NS |
|  | D2% | NS | NS | NS |
|  | V100% | 0.002 | NS | 0.012 |
